# Identification of RNA: 5-Methylcytosine Methyltransferases-Related Signature for Predicting Prognosis in Glioma

**DOI:** 10.3389/fonc.2020.01119

**Published:** 2020-08-19

**Authors:** Peng Wang, Miaojing Wu, Zewei Tu, Chuming Tao, Qing Hu, Kuangxun Li, Xingen Zhu, Kai Huang

**Affiliations:** ^1^Department of Neurosurgery, The Second Affiliated Hospital of Nanchang University, Nanchang, China; ^2^East China Institute of Digital Medical Engineering, Shangrao, China; ^3^Institute of Neuroscience, Nanchang University, Nanchang, China; ^4^School of Medicine, Queen Marry College, Nanchang University, Nanchang, China

**Keywords:** glioma, 5-methylcytosine methyltransferases, RNA modification, gene expression profile, prognostic signature

## Abstract

**Background:** Glioma is the most common primary intracranial tumor, accounting for the vast majority of intracranial malignant tumors. Aberrant expression of RNA:5-methylcytosine(m^5^C) methyltransferases have recently been the focus of research relating to the occurrence and progression of tumors. However, the prognostic value of RNA:m^5^C methyltransferases in glioma remains unclear. This study investigated RNA: m^5^C methyltransferase expression and defined its clinicopathological signature and prognostic value in gliomas.

**Methods:** We obtained the RNA-sequence and Clinicopathological data of RNA:m^5^C methyltransferases underlying gliomas from the Chinese Glioma Genome Atlas (CGGA) and The Cancer Genome Atlas (TCGA) datasets. We analyzed the expression of RNA:m^5^C methyltransferase genes in gliomas with different clinicopathological characteristics and identified different subtypes using Consensus clustering analysis. Gene Ontology (GO) and Gene Set Enrichment Analysis (GSEA) was used to annotate the function of these genes. Univariate Cox regression and the least absolute shrinkage and selection operator (LASSO) Cox regression algorithm analyses were performed to construct the risk signature. Kaplan-Meier method and Receiver operating characteristic (ROC) curves were used to assess the overall survival of glioma patients. Additionally, Cox proportional regression model analysis was developed to address the connections between the risk scores and clinical factors.

**Results:** We revealed the differential expression of RNA:m^5^C methyltransferase genes in gliomas with different clinicopathological features. Consensus clustering of RNA:m^5^C methyltransferases identified three clusters of gliomas with different prognostic and clinicopathological features. Meanwhile, functional annotations demonstrated that RNA:m^5^C methyltransferases were significantly associated with the malignant progression of gliomas. Thereafter, five RNA:m^5^C methyltransferase genes were screened to construct a risk signature that can be used to predict not only overall survival but also clinicopathological features in gliomas. ROC curves revealed the significant prognostic ability of this signature. In addition, Multivariate Cox regression analyses indicated that the risk score was an independent prognostic factor for glioma outcome.

**Conclusion:** We demonstrated the prognostic role of RNA:m^5^C methyltransferases in the initiation and progression of glioma. We have expanded on the understanding of the molecular mechanism involved, and provided a unique approach to predictive biomarkers and targeted therapy for gliomas.

## Introduction

Traditional epigenetic modifications, including DNA methylation, histone modification, and chromatin remodeling, target many biological processes that underlie the incidence and progression of cancer, including gliomas (1, 2). In recent years, with the advent and development of high-throughput sequencing technologies coupled with direct RNA-sequencing technologies, the focus has shifted to the study of epigenetic modifications of RNA (3, 4). Based on these sequencing technologies, published data reveals that RNA contains multiple dynamic modifications, among which the most studied are N6-methyladenosine (m^6^A), 5-methylcytosine (m^5^C), N1-methyladenosine (m^1^A), N7-methylguanosine(N^7^G), and ribose 2′-O-methylation, as well as pseudouridine (Ψ) and inosine (I) (5–9). The dynamic regulation and disorder of these RNA modifications are also significantly related to the occurrence, maintenance and progression of tumors (10, 11). Among these RNA modifications, the m^6^A was the first modification to be identified. Another well-studied modification of RNA is m^5^C (12). This post-transcriptional modification of m^5^C has been detected in most RNA species, including messenger RNAs (mRNA), mitochondrial ribosomal RNAs (rRNAs), transfer RNAs (tRNAs), enhancer RNAs (eRNAs), cytoplasmic RNAs, and non-coding RNAs (12–15). m^5^C methylation of RNA is catalyzed by the NOL1/NOP2/sun domain RNA methyltransferase family and the DNA methyltransferase homolog TRDMT1 (formerly known as the DNA methyltransferase member DNMT2) in eukaryotes, but the function of the binding proteins and demethylases remains unclear, while there is evidence to suggest that YBX1 might be the binding protein for m^5^C (11, 15–20). The cellular functions and modifications of these enzymes contribute to our understanding of the mechanism of m^5^C involvement in epigenetic inheritance related to various diseases, including tumors.

The NOL1/NOP2/sun domain RNA methyltransferase family consists of 7 members, namely NSUN1 (NOP2 nucleolar protein), NSUN2, NSUN3, NSUN4, NSUN5, NSUN6, and NSUN7. The biological function of these RNA:m^5^C methyltransferases and the modifications they induce have revealed their importance in several aspects of protein biosynthesis, cell proliferation, and differentiation, as well as on mitochondrial and nuclear gene expression (18, 20, 21). Moreover, it has become increasingly clear that aberrant expression of RNA:m^5^C methyltransferase may underlie the pathogenesis of several cancers. For instance, NSUN1, NSUN2, and NSUN4 were found to be overexpressed in a number of human cancers, including cancer of the breast, gallbladder, bladder, prostate, and cervix (16, 21–24).

Glioma is the most common primary intracranial tumor, accounting for the vast majority of intracranial malignant tumors, and is notorious for its high recurrence rate and resistance to treatment (2, 25, 26). In the past, extensive literature reports involving glioma and 5-methylcytosine have focused on DNA methylation, which could serve as biomarkers for diagnosis. With the rapid development of the Generation Sequencing technology, RNA modifications such as N6-methyladenosine (m^6^A) RNA methylation, especially its regulatory enzymes (“writers,” “erasers,” “readers”), have also emerged as important epigenetic mechanisms for the progression and malignancy of glioma. To date, no literature has reported the relationship between aberrant expression of these RNA:m^5^C methyltransferases and clinicopathological features, as well as the prognostic value of these methyltransferases with respect to gliomas. It may be useful to study the biological role of these enzymes as they have potential as therapeutic targets for the treatment of glioma.

In this study, we comprehensively studied the expression profiles of the NOL1/NOP2/sun domain RNA methyltransferase family, which are RNA:m^5^C methyltransferases, using the RNA sequencing data from the CGGA (*n* = 306) and TCGA (*n* = 616) datasets and aimed to investigate its prognostic value in glioma. We demonstrated the association between RNA and glioma malignant progression and constructed an RNA:m^5^C methyltransferase-related signature to evaluate the patients with glioma. Surprisingly, this gene signature can effectively predict the malignancy and prognosis of glioma patients.

## Materials and Methods

### Acquisition of Datasets and Human Tumor Samples

The CGGA RNA sequencing data [per kilobase of transcript per million mapped reads (RSEM)] and the relevant clinical information were downloaded from the CGGA data portal (http://www.cgga.org.cn/) as the training set. Similarly, the TCGA RNA sequencing data [fragments per kilobase of transcript per million mapped reads (FPKM)] and clinical information were downloaded from the TCGA data portal (https://www.cancergenome.nih.gov/) and used as a verification set. Moreover, the somatic mutation data from glioma patients were also downloaded from the TCGA data portal as MAF files. After the removal of samples with missing data on survival and WHO grade, we obtained 306 (CGGA dataset) and 616 (TCGA dataset) glioma patients. Thereafter, the CGGA and TCGA RNA sequencing data were normalized with Expectation-Maximization algorithm (log2 transformation) for the subsequent analysis. The clinical information for the CGGA and TCGA datasets was listed in Supplementary Table 1. Moreover, the seven RNA:m^5^C methyltransferases were screened according to the published literature (11, 17, 18, 20).

All human glioma tissue samples used in the study were obtained from patients who were operated on in The Second Affiliated Hospital of Nanchang University, Jiangxi, from 2015 to 2019. According to the World Health Organization classification, glioma samples were divided into four groups: Grade I, Grade II, Grade III, Grade IV. Twenty-five brain samples, including 9 high-grade glioma samples (WHO grade III, IV), 10 low-grade glioma samples (WHO grade II), and 6 non-neoplastic brain tissues (NBTs) were used as a control collected from the surgery of intractable epilepsy cases. The study was approved by the medical ethics committee of the Second Affiliated Hospital of Nanchang University and was performed in accordance with the approved guidelines. Informed consents were acquired from each glioma patient. The tumor tissues were immediately frozen and stored at −80°C.

### Consensus Clustering and Principal Components Analysis

To explore the function of gliomas' RNA:m^5^C methyltransferases-related genes, we identified three subtypes based on the RNA:m^5^C methyltransferase expression profiles of 306 patients with gliomas from the CGGA dataset using the R package “ConsensusClusterPlus” (50 iterations, 80% resampling rate Pearson correlation, http://www.bioconductor.org/) (27). The appropriate number of subtypes was calculated using cumulative distribution function (CDF) and consensus matrices. Thereafter, we used principal components analysis (PCA) to detect differential gene expression amongst the three subtypes using R package pca3d and rgl (28).

### Biological Functional Analysis

Function and interaction of the seven RNA:m^5^C methyltransferases were predicted using the String website (https://string-db.org/) and the R package “corrplot.” Gene Ontology (GO) analysis was performed with Metascape (http://metascape.org/) to annotate the function of differentially expressed genes in the different subtypes. Furthermore, the Kyoto Encyclopedia of Genes and Genomes (KEGG) pathway enrichment was performed using Database for Annotation, Visualization and Integrated Discovery (DAVID; https://david.ncifcrf.gov/; version 6.8) and visualized in the imageGP website (http://www.ehbio.com/ImageGP/). Gene set enrichment analysis (GSEA; version 4.0.3) was performed using the JAVA program and downloaded from the website (http://software.broadinstitute.org/gsea/). The hallmark gene set (h.all.v6.0.symbols.gmt) was also downloaded. Thereafter, the hallmark gene set was determined to be significantly enriched following normalization (*p* < 0.05) and a false discovery rate (*FDR* < 0.25).

### Construction and Evaluation of Risk Score

Univariate Cox regression analysis was performed to identify the genes significantly related to survival (*P* < 0.05). Thereafter, six RNA:m^5^C methyltransferases (NOP2, NSUN2, NSUN4, NSUN5, NSUN6, NUSN7) were screened with the LASSO multivariate Cox regression algorithm using the R package “glmnet” (version 3.0). Finally, the signature genes and coefficients in the risk score signature were constructed based on the most suitable penalty parameter λ. The risk score formula we used was:

$$\begin{array}{l} Risk\;score=\overset{n}{\underset{i=1}{\sum }}\left(Coef_{i}*Exp_{i}\right) \end{array}$$

where *Coef*_*i*_ is the coefficient and *Exp*_*i*_ is the normalized expression of each signature gene. The risk score system of five RNA:m^5^C methyltransferases (NOP2, NSUN4, NSUN5, NSUN6, NUSN7) was constructed in the CGGA dataset, and evaluated with the TCGA dataset. Patients were ranked into high-risk and low-risk groups using the median risk score. Moreover, the genomic alterations of these glioma patients were analyzed using the R package “Maftools” (29).

### The Human Protein Atlas

The immunohistochemistry pathological specimen results from the prognostic RNA:m^5^C methyltransferases were downloaded from The Human Protein Atlas (https://www.proteinatlas.org/). Staining intensity, quantity and patients' information can be inquired about online.

### Quantitative Real-Time Polymerase Chain Reaction (qRT-PCR)

To assess the mRNA expression levels of RNA:m^5^C methyltransferases in glioma patients we used trizol reagent (Invitrogen, Carlsbad, CA), extracted total RNA from brain tissues and the total RNA (2 μg) was reverse transcribed into cDNA, according to manufacturer's instructions. The qRT-PCR was performed on The LightCycler® 480 Real-Time PCR System. The expression level of genes was analyzed and normalized to GAPDH and the 2^−ΔΔCT^ method was performed to evaluate the fold change in gene expression. The following primer sequences (Shanghai GenePharma Co., Ltd.): NOP2 forward 5′-AAATGGGAGAAGGTGGCGTC-3′, and reverse 5′-CTCTCGGACATTAACCCGCA-3′; NSUN4 forward 5′-ATGTGCCCTGTACCACAGAC-3′, and reverse 5′-GGTAAGAGCTTCCAGTCCCC-3′; NSUN5 forward 5′-TGCTTCTCAGAGCCAACGAA-3′, and reverse 5′-CTTCCAGACTGTCTCACTGCAT-3′; NSUN6 forward 5′-CTCGGTGACAGGGAGGTCTA-3′, and reverse 5′-AGGCATCTCTCATGGTGAGG-3′; NSUN7 forward 5′-TCAGCTCCAGATTGCGATTC-3′, and reverse 5′-CCCTCGTGCCTTAGGATCAC-3′.

### Statistical Analysis

The One-way ANOVA and R packages “limma” were used to compare the expression level of RNA:m^5^C methyltransferases in gliomas with different WHO grades and IDH status. The *t*-tests were performed to contrast the expression levels in gliomas for clinicopathological features. A chi-square test was conducted to compare the distribution of clinical and molecular pathological features between the different groups stratified by risk scores. Univariate, multivariate, LASSO Cox regression and Kaplan-Meier analyses were performed to construct and evaluate the risk signature using the R packages “glmnet” and “survival” (30, 31). Roc curve analysis was performed to predict the OS of glioma patients using the R package “survivalROC.” All statistical analyses were two-sided and conducted using R software (version 3.6.1; https://www.r-project.org/) and SPSS (version 26). *P* < 0.05 was considered statistically significant.

## Results

### The Relationship Between the Aberrant Expression of RNA:m^5^C Methyltransferases and Clinicopathological Characteristics of Gliomas

**Figure 1:**
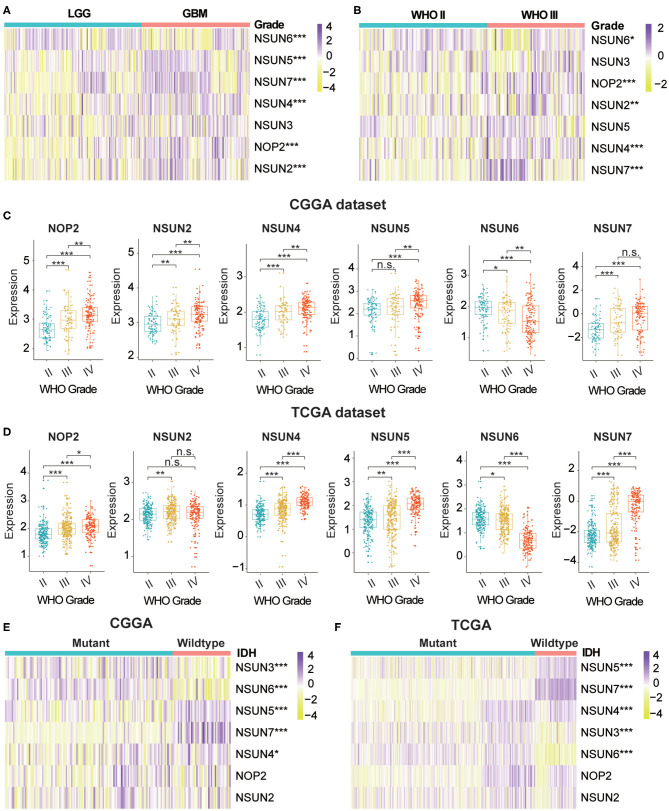
The relationship between the aberrant expression of RNA:m^5^C methyltransferases and clinicopathological characteristics of gliomas in the CGGA and TCGA datasets. **(A,B)** The differential expression of seven RNA:m^5^C methyltransferases with different WHO grades in gliomas. **(C,D)** The significant differential expression of RNA:m^5^C methyltransferases stratified by WHO grade in gliomas. **(E,F)** The differential expression of RNA:m^5^C methyltransferases stratified by IDH-mutant status in low-grade gliomas. Significance: *P < 0.05, **P < 0.01, ***P < 0.001.

m^5^C modifications of RNA play an important role in the occurrence and progression of tumors and influence many biological functions. We comprehensively studied the relationship between each RNA:m^5^C methyltransferase and the clinicopathological characteristics of gliomas, for example, WHO grades and isocitrate dehydrogenase (*IDH*)-mutant status. The relationship between aberrant expression of RNA:m^5^C methyltransferases and WHO grades are shown using heatmaps (Figures 1A,B). As shown in the heatmaps, the expression of almost all RNA:m^5^C methyltransferases was significantly correlated with WHO grade. The significant differentially expressed RNA:m^5^C methyltransferases include NOP2, NSUN2, NSUN4, NSUN5, NSUN6, and NSUN7. Thereafter we quantitatively analyzed the expression of these differentially expressed genes in the CGGA dataset (Figure 1C) and verified our findings using the TCGA dataset (Figure 1D). As shown in the figure, NOP2, NSUN2, NSUN4, NSUN5, and NSUN7 were up-regulated and NSUN6 was down-regulated with an increase of WHO grade.

Mutation of the *IDH* gene has been reported in gliomas, particularly in low-grade gliomas (LGGs), and the prognostic value of the mutation has been confirmed by many authors in the literature. Based on these findings, we investigated the relationship between aberrant expression of RNA:m^5^C methyltransferases and *IDH*-mutant status in LGGs. The results showed different expression of NSUN3, NSUN4, NSUN5, NSUN6, and NSUN7 between *IDH*-mutant and *IDH*-wildtype status in both CGGA (Figure 1E) and TCGA (Figure 1F) datasets. In patients harboring *IDH* mutants, the expression of NSUN4, NSUN5, and NSUN7 increased, while the expression of NSUN3 and NUSN6 decreased. We also studied the expression of RNA:m^5^C methyltransferases in glioblastomas (GBM) stratified by *IDH* mutant status, and findings indicated that NSUN5, NSUN6, and NSUN7 were still differentially expressed (Supplementary Figures 1A,B).

In addition, we predicted the gene mutation frequencies of seven RNA:m^5^ methyltransferases in the cBioPortal of the Cancer Genomics Database and verified these findings using the TCGA dataset, resulting in that mutations of these RNA:m^5^C methyltransferases were rare (Supplementary Figure 1C and Supplementary Table 4). Even for the top-ranked RNA:m^5^C methyltransferases like NOP2, the mutation frequencies were only 3%. It demonstrated that the aberrant expression of RNA:m^5^C methyltransferases may not be generated by a genetic mutation.

### Interaction and Unsupervised Consensus Analysis of These RNA:m^5^C Methyltransferases

**Figure 2:**
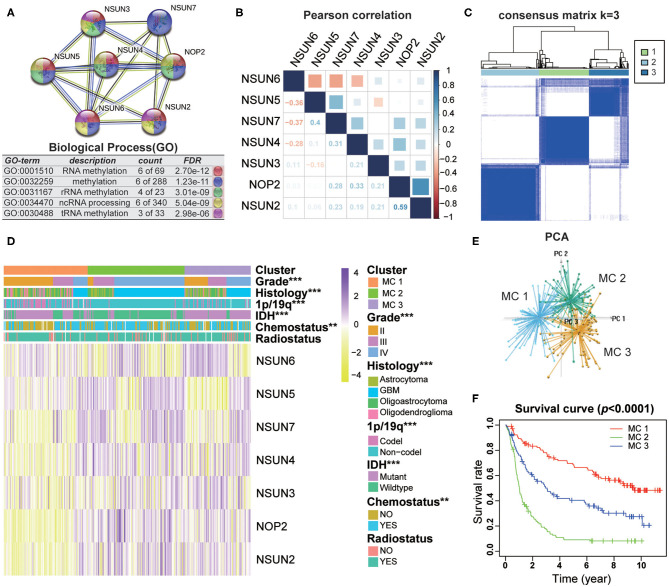
Interaction and unsupervised consensus analysis of selected RNA:m^5^C methyltransferases. **(A)** The function and interaction of seven RNA:m^5^C methyltransferases. **(B)** Pearson correlation analysis of seven RNA:m^5^C methyltransferase expression profiles in the CGGA dataset. **(C)** Consensus clustering matrix for the most suitable k = 3. **(D)** The relationship between the RNA:m^5^C methyltransferase expression profiles of these three subtypes and clinicopathological features of gliomas. **(E)** Principal component analysis (3D) of the CGGA RNA-sequence profiles. **(F)** Kaplan-Meier overall survival curves for the glioma patients of three subtypes in the CGGA dataset. Significance: *P < 0.05, **P < 0.01, ***P < 0.001.

To investigate the close connection between RNA:m^5^C methyltransferases and the clinicopathological features of gliomas, we systematically investigated the function, interaction, and correlation of RNA:m^5^C methyltransferases. We found that all the RNA:m^5^C methyltransferase genes were involved in various types of methylation, among which NOP2, NSUN3, NSUN4, and NSUN5 were mainly involved in rRNA methylation and NSUN2, NSUN6, as well as NSUN3, participated in tRNA methylation. Their function and interaction were supported by text mining and co-expression (Figure 2A). Thereafter, a Pearson correlation analysis was performed to study the expression profile of seven RNA:m^5^C methyltransferases in the CGGA (Figure 2B) and TCGA (Supplementary Figure 2A) datasets. The expression of NOP2, NSUN2, NSUN4, NSUN5, and NSUN7 were positively correlated in gliomas. Whereas the expression of NSUN6 was significantly negatively correlated with NSUN4, NSUN5 as well as NSUN7. These results were consistent with the quantitative analysis of RNA:m^5^C methyltransferases expression profile in gliomas, indicating that the expression levels of NOP2, NSUN2, NSUN4, NSUN5, and NSUN7 were shown to be positively correlated with the malignant progression of glioma, while the expression of NSUN6 were negatively correlated with glioma.

Based on the RNA:m^5^C methyltransferase expression profiles of 306 patients with gliomas in the CGGA dataset, we used unsupervised consensus clustering analysis to identify three subtypes, namely MC1, MC2, and MC3 (Figure 2C). Clearly, k = 3 seemed to be a relatively stable distinction of the samples in the CGGA dataset with clustering stability increasing from k = 2 to k = 9 (Supplementary Figures 2B–E). Furthermore, the PCA analysis was used to compare the transcription profiles of these three subgroups. The results showed that they could be adequately divided into three distinct clusters (Figure 2E). Next, we investigated the relationship between the RNA:m^5^C methyltransferase expression profiles of these three subtypes and clinicopathological features of gliomas (Figure 2D). In these three subtypes, MC2 subtype compared to MC3 subtype and MC3 subtype compared to MC1 subtype were significantly correlated with a higher grade (*P* < 0.0001), *IDH*-wildtype status (*P* < 0.0001), 1p/19q-noncodel status (*P* < 0.0001), older average age at diagnosis (*P* < 0.0001), and receipt of additional chemotherapy (*P* < 0.01) (Supplementary Table 2). Moreover, we found significant differences in overall survival between the three groups (*P* < 0.0001). The survival of patients who fell into the MC2 subtype was obviously shorter than for the other two subtypes (Figure 2F). Moreover, the unsupervised consensus analysis of these RNA:m5C methyltransferases in the TCGA dataset were consistent with the results of the CGGA dataset (Supplementary Figures 2F–J). The results above indicating that consensus clustering of RNA:m^5^C methyltransferases could identify subtypes with different clinicopathological features and prognosis in gliomas.

### Functional Annotation of The Subtypes

**Figure 3:**
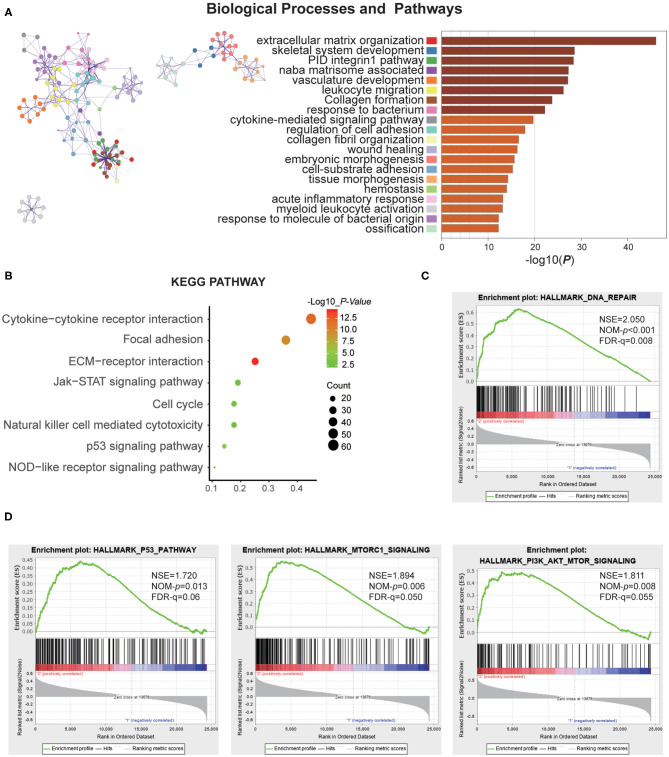
Function annotation of specific genes of the MC2 subtype. **(A)** Network and bar chart of 20 significantly enriched biological processes of up-regulated genes in the MC2 subtype. Each enriched node is presented in a different color. **(B)** KEGG pathway analysis of up-regulated genes in the MC2 subtype. **(C,D)** GSEA analysis of the MC2 subtype showed enrichment for various hallmarks of tumors.

To investigate the different clinicopathological features and overall survival rates of the three groups in gliomas, we annotated the biological processes of specific genes associated with the MC2 subtypes. A comparison was performed with the other two groups, in which 664 genes were up-regulated (log FC > 1.5, normalized *P* < 0.01 and FDR = 0.05) and 645 genes were down-regulated (log FC < −1.5 and normalized *P* < 0.01, FDR = 0.05) in MC2 relative to MC1 and MC3 subtypes. Go analysis of the up-regulated genes revealed that “extracellular matrix organization,” “vasculature development,” “epithelial cell proliferation,” “cell-substrate adhesion,” and “cellular response to tumor necrosis factor” were enriched in biological processes and pathways which might be highly correlated with malignant progression of gliomas. The top 20 significantly enriched biological processes were shown in the figure (Figure 3A). The KEGG pathway analysis further revealed that these genes were also notably associated with tumor-relevant signaling pathways, for example, ECM-receptor interaction, Jak-STAT signaling pathway, and P53 signaling pathway, amongst others (Figure 3B).

Moreover, gene set enrichment analysis (GSEA) was performed to investigate the hallmarks of tumors in the MC2 subtype. The results indicated that the tumor hallmarks, such as P53 pathway (NES = 1.720, *P* = 0.013), P13K/AKT/mTOR signaling (NES = 1.811, *P* = 0.008), DNA repair (NES = 2.050, *P* < 0.001), and MTORC1 signaling (NES = 1.894, *P* = 0.006) (Figures 3C,D) were enriched in MC2 subtype. Combined with the above analysis, the subtypes identified by RNA:m^5^C methyltransferases were significantly associated with the malignant progression of gliomas.

### Prognostic Value of RNA:m^5^C Methyltransferases and Construction of The Risk Score Signature by Five RNA:m^5^C Methyltransferase Genes

**Figure 4:**
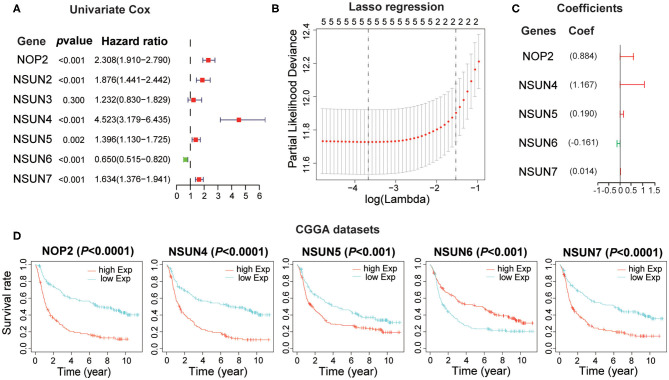
Construction of the risk score signature using five RNA:m^5^C methyltransferase genes. **(A)** Univariate Cox regression analysis of seven RNA:m^5^C methyltransferases in the CGGA dataset. **(B,C)** Identification of five prognostic genes in the CGGA dataset and the coefficients constructed using the LASSO method. **(D)** Kaplan-Meier overall survival curves for each prognostic gene in the CGGA dataset.

Based on the relationship between RNA:m^5^C methyltransferases and the malignant progression of gliomas, we further attempted to explore the prognostic role of RNA:m^5^C methyltransferases in gliomas by a univariate survival analysis using Cox proportional hazards models of expression levels in the CGGA dataset, which was set up as a training dataset. We obtained six genes associated with prognosis (*P* < 0.01), among which NOP2, NSUN2, NSUN4, NSUN5, and NSUN7 acted as risk factors (HR > 1), and NSUN6 played a protective role (HR < 1) in gliomas (Figure 4A). To improve the robustness of the six RNA:m^5^C methyltransferases, these genes were selected to conduct an additional analysis by the least absolute shrinkage and selection operator (LASSO) Cox regression algorithm in the CGGA dataset (Figure 4B). Five genes of RNA:m^5^C methyltransferase genes and coefficients (Figure 4C) were screened to construct the risk score signature, and the formula for the risk score is as follows: 0.884 × (expression value of NOP2) + 1.167 × (expression value of NSUN4) + 0.190 × (expression value of NSUN5) + (−0.161) × (expression value of NSUN6) + 0.014 × (expression value of NSUN7) both in the training dataset (CGGA) and the verification dataset (TCGA). In this risk score signature, four genes (NOP2, NSUN4, NSUN5, and NSUN7) were cancer-promoting and NSUN6 was a cancer-suppressor gene. To better understand the role of these five prognostic genes in glioma, K-M analysis was performed both in the CGGA dataset and TCGA dataset in which samples were classified by high or low expression according to the median gene expression level. All five of these RNA:m^5^C methyltransferase genes were significantly correlated with OS (*P* < 0.0001) (Figure 4D and Supplementary Figure 3A).

### The Power of the Prognostic Value of the Risk Score Signature in Glioma

**Figure 5:**
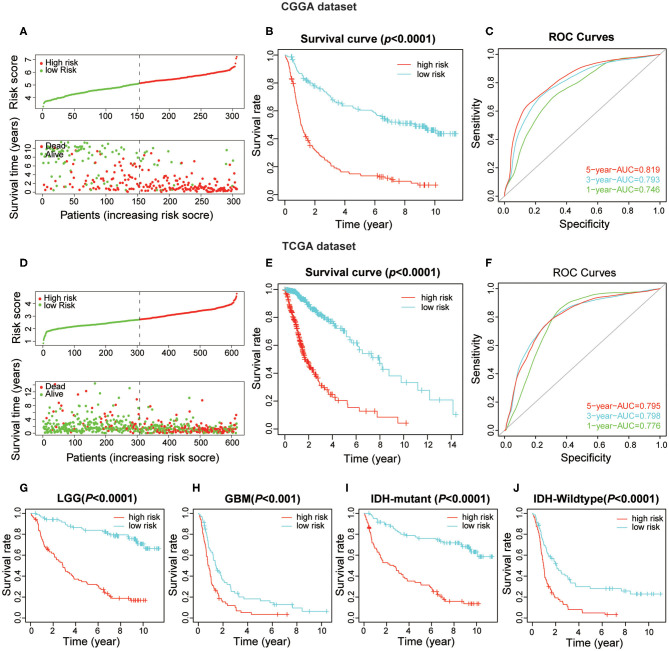
The prognostic value of the risk score signature both in CGGA and TCGA datasets. **(A)** The patients with high-risk scores were correlated with a higher death rate and shorter survival time in gliomas. **(B)** Kaplan-Meier overall survival curves for patient data in the CGGA dataset. **(C)** The area under the curve (AUC) of ROC curves were 0.819, 0.793, and 0.746 in predicting 5-, 3-, and 1-year OS events from the CGGA dataset, respectively. **(D–F)** Validation of the risk score signature in the TCGA dataset using the same analysis. **(G–J)** Kaplan-Meier overall survival curves for patients in the CGGA dataset stratified by WHO grade and IDH-mutant status.

To obtain the predictive effect of the risk score signature for clinical outcomes in patients with gliomas, the median of all patients' scores was used as a standard, and the data were divided into high- and low-risk groups both in the CGGA and TCGA datasets. The analyses indicated that the number of patients who died increased significantly as the risk score increased (Figures 5A,D). In addition, there was a significant difference in overall survival (*P* < 0.0001) between the high-risk group and low-risk group (Figures 5B,E). Thereafter, the ROC curve analyses at 1-, 3-, and 5-years for prognostic risk scores were performed to test the predictive efficiency of the risk signature. The results showed that the risk score had high accuracy [the area under the curve (AUC) of all results in the ROC curve were >0.750] in distinguishing the OS of gliomas (Figures 5C,F). Moreover, we performed further studies on the prognostic value of this signature in glioma patients stratified by WHO grade and *IDH*-mutant status. The results revealed that the risk score signature could be used to divide glioma patients, in the CGGA dataset, into two distinct prognosis groups with different WHO grade subtypes (LGG and GBM) and *IDH*-mutant status subtypes (Figures 5G–J). The consensus results of the risk score signature were also obtained using the TCGA dataset (Supplementary Figures 3B–E). From the comprehensive analyses above, we concluded that the prognostic efficacy of the risk score was accurate and stable.

### The Interrelation of The Risk Scores and Clinicopathological Characteristics in Patients With Gliomas

**Figure 6:**
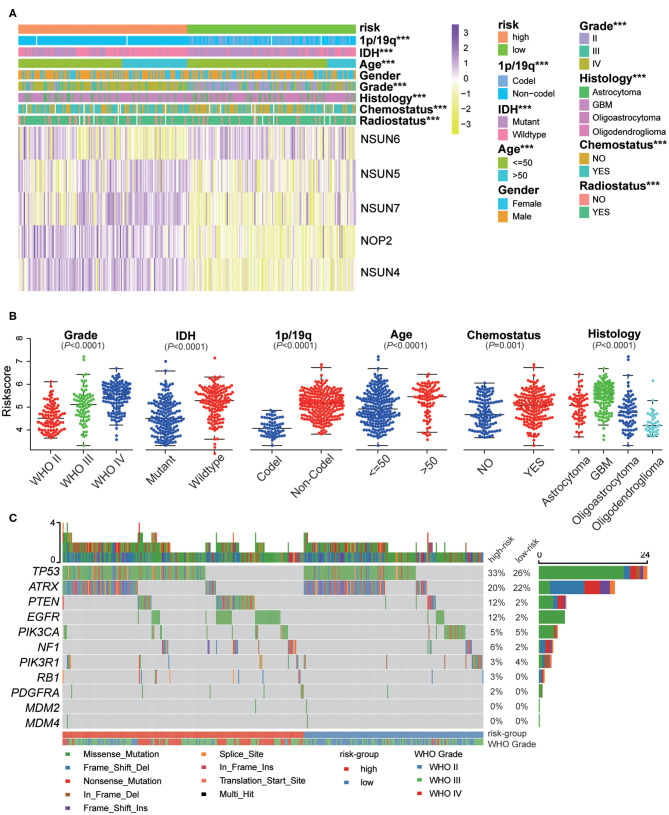
The interrelation of the risk scores and clinicopathological characteristics in gliomas, and the mutational landscape of tumor driver-gene alterations. **(A)** The relationship between five RNA:m^5^C methyltransferase expression profiles stratified by risk score and clinicopathological features of gliomas. **(B)** The relationship between the risk scores and each clinicopathological characteristic. **(C)** The mutational landscape of tumor driver-gene alterations between low- and high-risk patients with gliomas. Significance: *P < 0.05, **P < 0.01, ***P < 0.001.

The expression of the five screened RNA:m^5^C methyltransferases in low- and high-risk patients in the CGGA dataset are represented by heatmaps (Figure 6A). We found statistically significant differences between the low-risk and high-risk groups both in the CGGA and TCGA datasets, based on WHO grade (*P* < 0.0001), histology (*P* < 0.0001), *IDH* status (*P* < 0.0001), 1p/19q status (*P* < 0.0001), age (*P* < 0.0001), and receipt of additional chemotherapy (*P* < 0.0001) (Supplementary Table 3). Thereafter, we quantitatively analyzed the risk scores in glioma to investigate the association between the risk scores and each clinicopathological characteristic. As shown in the figure, the risk scores were significantly different in these groups with the CGGA dataset (Figure 6B) compartmentalized by WHO grade, *IDH* status, 1p/19q status, histology, age and receipt of additional chemotherapy, and were verified in the TCGA dataset (Supplementary Figures 4A–G). The results demonstrated that the risk scores identified by five RNA:m^5^C methyltransferases were significantly correlated with malignancy of glioma. Moreover, considering the importance of reported glioma-associated driver-gene alterations in glioma initiation and progression, including *ATRX*, P53 pathway (*TP53, MDM2, MDM4*), RB pathway (*CDK4, CDK6, CCND2, CDKNA/B, RB1*), and P13K/RTK pathway (*PIK3CA, PIK3R1, PTEN, EGFR, PDGFRA, NF1*), we obtained the somatic mutation data from the TCGA database (32, 33). The mutational landscape of tumor driver-gene alterations between low- and high-risk patients with gliomas was rendered as a waterfall plot (Figure 6C). The high-risk patients were characterized by more frequent alterations in tumor driver-genes than low-risk patients. This means high-risk patients harbored more progressive cancer. Combining the above results, the risk scores can predict not only overall survival but also clinicopathological features.

**Figure 7:**
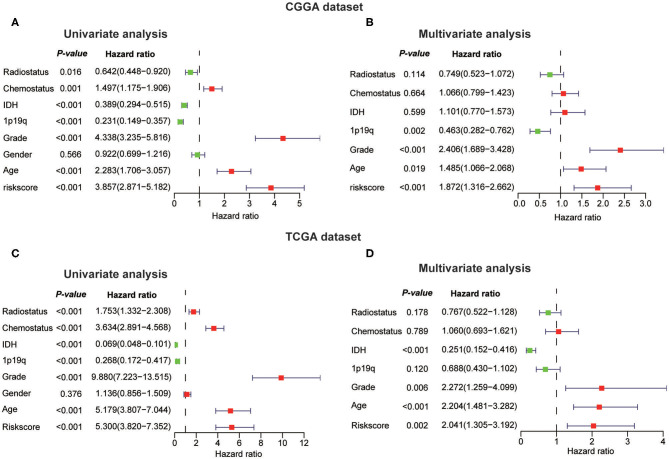
Univariate and multivariate Cox regression analyses of eight clinicopathological features and biological function analyses. **(A,B)** The risk score was an independent prognostic factor in the CGGA dataset. **(C,D)** The risk score was an independent prognostic factor in the TCGA dataset.

Next, we investigated whether this risk score was an independent prognostic factor based on eight clinicopathological features. Univariate and multivariate Cox regression analyses were performed with the CGGA dataset. We observed that the risk score, age, WHO grade, *IDH* status, 1p/19q codel status, chemotherapy status, and radiotherapy status were significantly correlated with prognosis using the univariate analysis (Figure 7A). Multivariate analysis based on the above factors was performed and the risk score remained strongly associated with the OS (*P* < 0.001, Figure 7B). In the verification dataset (TCGA) we obtained similar results, including the same factors in the multivariate analysis; the risk score also remained strongly associated with the OS (*P* = 0.002, Figures 7C,D). The consensus results demonstrated that the risk score constructed by RNA:m^5^C methyltransferases was a powerful and independent prognostic factor for glioma outcome.

### The mRNA and Protein Expression Patterns of Five Prognostic RNA:m^5^C Methyltransferases in Gliomas

**Figure 8:**
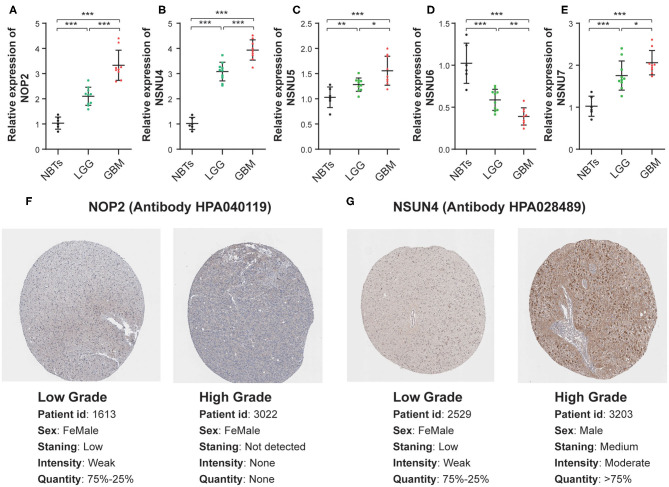
The mRNA and protein expression patterns of five prognostic RNA:m^5^C methyltransferases in gliomas**. (A–E)** The mRNA expression patterns of five prognostic RNA:m^5^C methyltransferases in non-neoplastic brain tissues and glioma tissues. **(F,G)** Comparison of protein expression in immunohistochemical specimens of NOP2 and NSUN4 in glioma. Significance: *P < 0.05, **P < 0.01, ***P < 0.001.

To assess the genes that are significant for construction of the risk score signature, we performed the qRT-PCR assay and acquired the immunohistochemistry pathological specimen data from the website of The Human Protein Atlas. As shown in the figure, all five RNA:m^5^C methyltransferase genes, NOP2, NSUN4, NSUN5, NSUN6, and NSUN7, were differentially expressed between normal brain tissues, low grade and high grade glioma tissues (Figures 8A–E). Furthermore, as shown in the figure, we found that the protein expression of NOP2 and NSUN4 in high-grade glioma tissues were much higher than those in low-grade glioma tissues (Figures 8F,G). However, the protein expression pattern of NSUN5, NSUN6, and NSUN7 was not presented here due to lack of immunohistochemistry images.

## Discussion

The important role played by aberrant RNA epigenetic modifications in tumorigenesis and tumor progression, as well as patient prognosis, has been increasingly demonstrated. This suggests that epigenetic regulators have a potential application in glioma diagnosis and prognosis. In the past, extensive literature reports involving glioma and 5-methylcytosine have focused on DNA methylation, which could serve as ideal biomarkers for cancer diagnosis (34–36). In this research, we focus on RNA epigenetic modifications, investigating the aberrant expression of m^5^C methyltransferase of RNA to explore whether RNA:m^5^C methyltransferases also participated in glioma initiation and progression as well as being correlated with glioma prognosis. By analyzing expression profiles of RNA:m^5^C methyltransferases from two open-access databases (TCGA and CGGA datasets), we identified three subtypes with different clinicopathological characteristics and prognoses. Moreover, the subtypes were closely correlated with tumor-related hallmarks, biological processes, and signaling pathways. Based on the features of RNA:m^5^C methyltransferases, we constructed a related risk score algorithm that divided glioma patients into high- and low-risk groups to precisely predict clinical outcomes of glioma patients. Furthermore, univariate and multivariate Cox regression analyses were performed to demonstrate that it was an independent prognostic factor for glioma patients, in addition to the WHO grade and *IDH*-mutant status as well as age at diagnosis. The signature-based on five RNA:m^5^C methyltransferases can serve as a potent prognostic signature and effectively stratify glioma patients based on risk scores and provide new insight into targeted therapy.

Of these seven RNA:m^5^C methyltransferases, several genes have been reported to be involved in tumor progression across malignancies. According to the latest literature, NSUN5 plays an important role in ribosomal RNA cellular transformation and protein translation. Furthermore, NSUN5 epigenetic inactivation was associated with a better prognosis for glioma patients (37, 38). NSUN2 is the most studied RNA:m^5^C methyltransferases, and involved in the methylation of various tRNAs and mRNAs. NSUN2 can stabilize the mitotic spindle to promote tumor cell proliferation and was used to identify several targets reported in gallbladder carcinoma, bladder cancer and several tumors (16, 21, 22, 39). NSUN1 (also named NOP2, p120) encodes a protein specific to the nucleolus. It plays an important function in the synthesis of ribosomes and cell cycle of tumor proliferation (24, 40). NSUN4 acts as cancer risk loci (Breast, Ovarian, and Prostate Cancer), and the identified MTERF4-NSUN4 axis plays a unique role in the biogenesis of mitochondrial ribosomes (23, 41, 42). With respect to NSUN6 and NUSN7, there have been no reports on tumors and related mechanisms, and further studies are required. In this study, we found that the expression of the genes described above was increased as the WHO grade and age at diagnosis increased as well as with *IDH*-mutant status, indicating that the expression levels of these genes are highly correlated with malignant progression of gliomas. To the best of our knowledge, this study is the first to link these genes to the prognosis and clinical characteristics of gliomas, providing indications for further study of the molecular mechanisms involved.

For a comprehensive analysis and validation, we tested our m^5^C RNA methyltransferase-related signature in a training set (CGGA) and a verification set (TCGA), respectively, and found their prognostic value in predicting OS. The results of subsequent multivariate Cox regression analyses eliminated four variables other than grade and age, although among which *IDH* had been proved to be an effective prognostic indicator (43, 44). This may be due to a deficiency in clinical information in the sample set, resulting in conflicting or meaningless results. In addition, we explored the mutational landscape of tumor driver-gene alterations between low-risk and high-risk patients with gliomas. The results of obviously different driver-gene alterations associated with risk further prove the accuracy of this signature. Furthermore, the differences in biological process analysis revealed that RNA:m^5^C methyltransferases were mainly involved in the regulation of cell cycle and cell proliferation and angiogenesis. Meanwhile, considering the protein translation regulatory mechanism of NSUN4 and NSUN5 targeting ribosomal RNA and the effects of NOP2, NSUN2 on cell proliferation in other tumors, this may provide a new direction to determine the mechanism of malignant glioma progression in the context of these methyltransferases. It is unclear whether RNA:m^5^C methyltransferases have potential clinical value for drug therapy, and this requires further study.

We used a combination of multi-omics, multi-dataset, and multi-ethnics analyses to demonstrate the robustness of our results. However, this study still has various limitations and requires further optimization. Additional fundamental experiments are needed to reveal the molecular mechanism of m^5^C RNA methyltransferases in glioma progression and the predictive efficiency of this signature needs to be tested for clinical application.

## Conclusion

We first revealed the significant correlation between the aberrant expression of RNA:m^5^C methyltransferases and malignant progression of gliomas. An m^5^C RNA methyltransferase-related signature was built which could effectively stratify glioma patients into high risk and low risk so as to accurately predict their survival. This study has significance for the role of RNA:m^5^C methyltransferases to enable us to enhance our understanding of the molecular mechanisms involved in the initiation and progression of gliomas and provides a unique approach to the discovery of predictive biomarkers and the selection of targeted therapy for the treatment of gliomas.

## Data Availability Statement

Publicly available datasets were analyzed in this study. This data can be found here: (http://www.cgga.org.cn/, https://www.cancergenome.nih.gov/).

## Ethics Statement

The studies involving human participants were reviewed and approved by the medical ethics committee of the Second Affiliated Hospital of Nanchang University. The patients/participants provided their written informed consent to participate in this study.

## Author Contributions

PW and KH designed the research. PW, KH, CT, and KL contributed to the data collection and analysis, figures and tables, and were involved in manuscript writing. ZT and PW contributed to the conducted the experiments. XZ and KH performed the correction of the language and revision. All authors proofread and approved the final manuscript.

## Conflict of Interest

The authors declare that the research was conducted in the absence of any commercial or financial relationships that could be construed as a potential conflict of interest.
