# Supplementary material for: Identification of RNA: 5-Methylcytosine Methyltransferases-Related Signature for Predicting Prognosis in Glioma
Source: Front Oncol. 2020 Aug 19;10:1119. doi: 10.3389/fonc.2020.01119 (PMC7466433; doi:10.3389/fonc.2020.01119)
Supplement: Supplementary file 1 [file Data_Sheet_1.pdf]

Supplementary Material

1 Supplementary Data

None

2 Supplementary Figures and Tables

2.1 Supplementary Figures

Supplementary Figure S1. The genetic alteration and differential expression of RNA:m^{5}C methyltransferases. (**A**-**B)** The differential expression of seven RNA:m^{5}C methyltransferases with different IDH-mutant status in glioblastoma. (**C**) The gene mutation frequencies of seven RNA:m^{5}C methyltransferases.

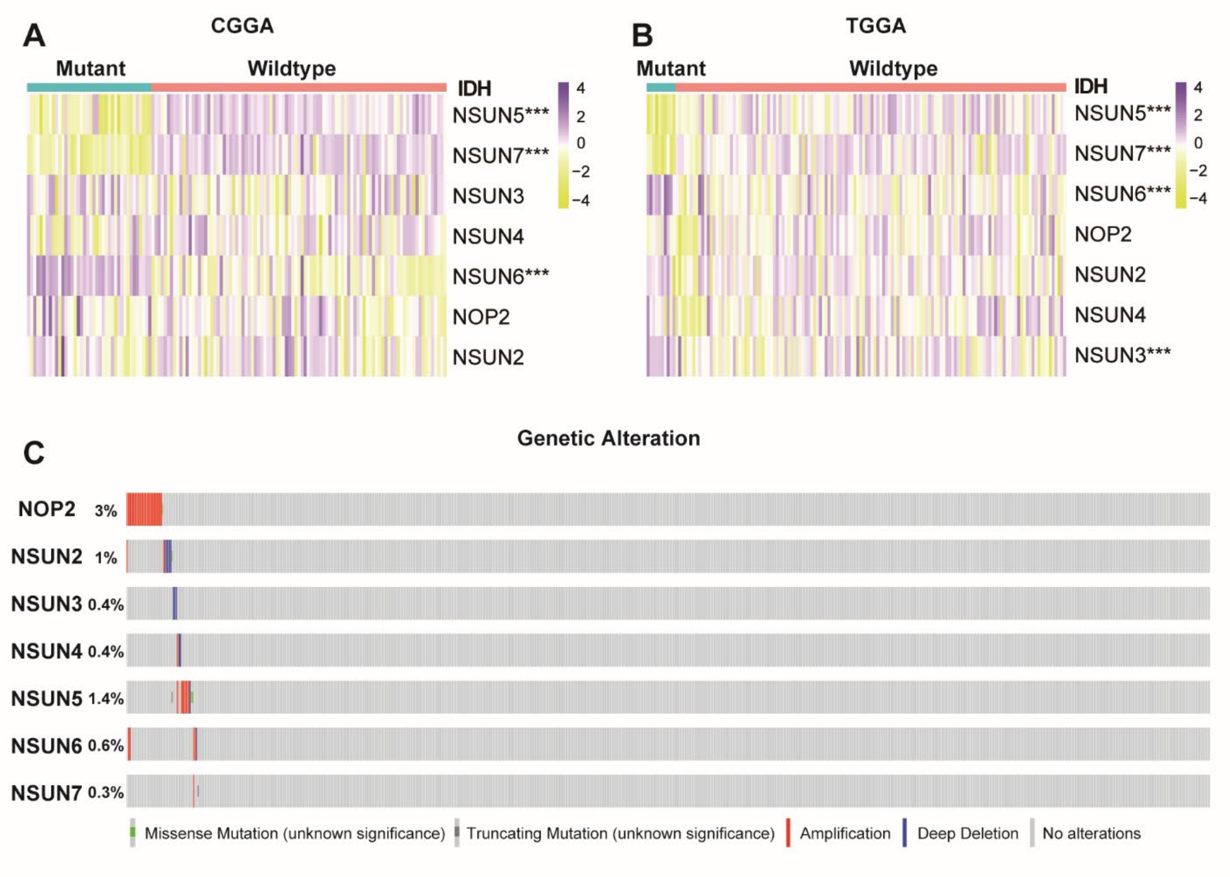

Supplementary Figure S2. Pearson correlation and unsupervised consensus analysis of selected RNA:m^{5}C methyltransferases. (**A**) Pearson correlation analysis of seven RNA:m^{5}C methyltransferase expression profiles in the TCGA dataset. (**B-C**) Consensus clustering cumulative distribution function for k=2 to 9. Relative change in area under CDF for k=2 to 9. (**D-E**) Consensus clustering matrix for k=2 and k=4.

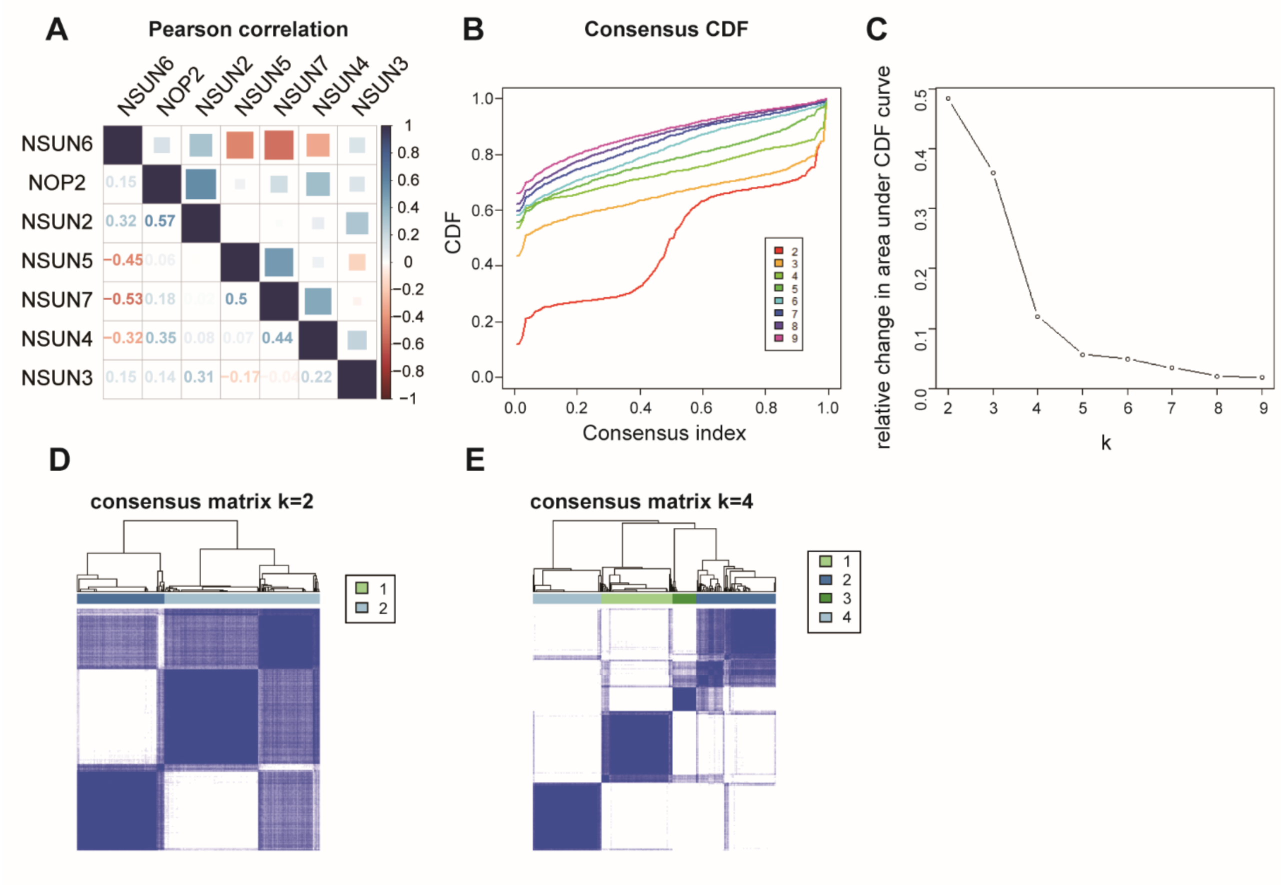

Supplementary Figure S3. Kaplan-Meier overall survival curves for patients in the TCGA dataset. (**A**) Kaplan-Meier overall survival curves for each prognostic gene in the TCGA dataset. (B-C), Kaplan-Meier overall survival curves for patients in the TGGA dataset stratified by WHO grade. (**D-E**) Kaplan-Meier overall survival curves for patients in the TGGA dataset stratified by IDH-mutant status.

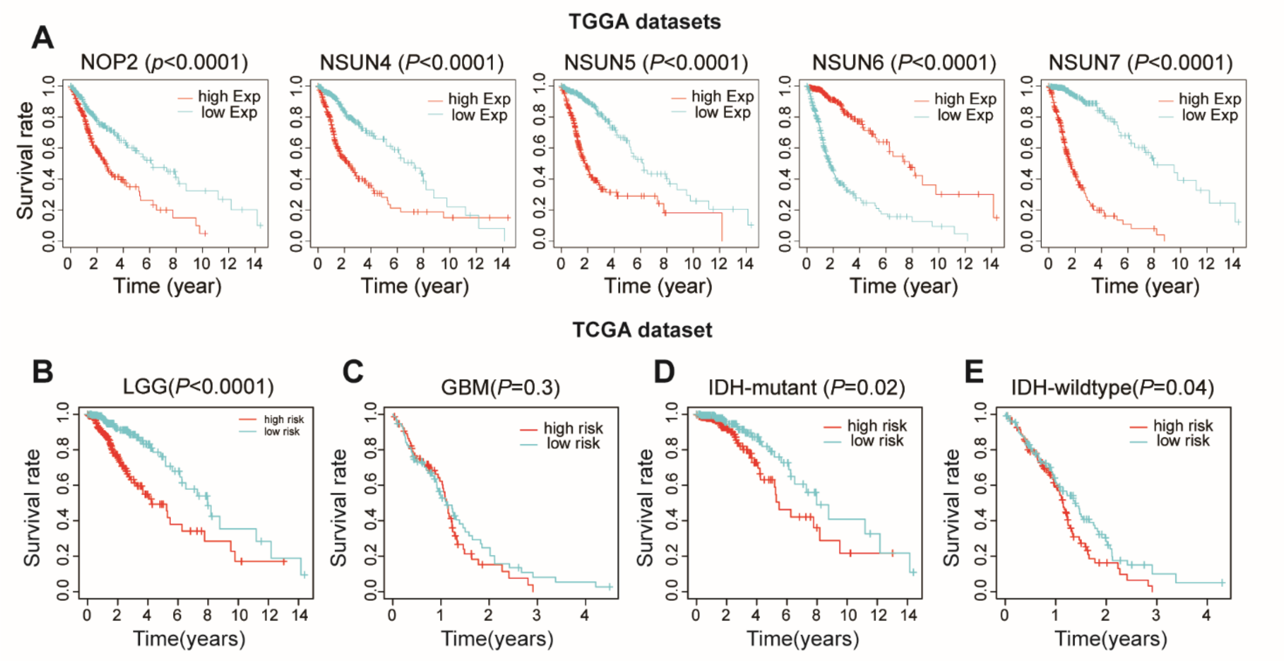

Supplementary Figure S4. A-G, The relationship between the risk scores and each clinicopathological characteristic in TCGA dataset.

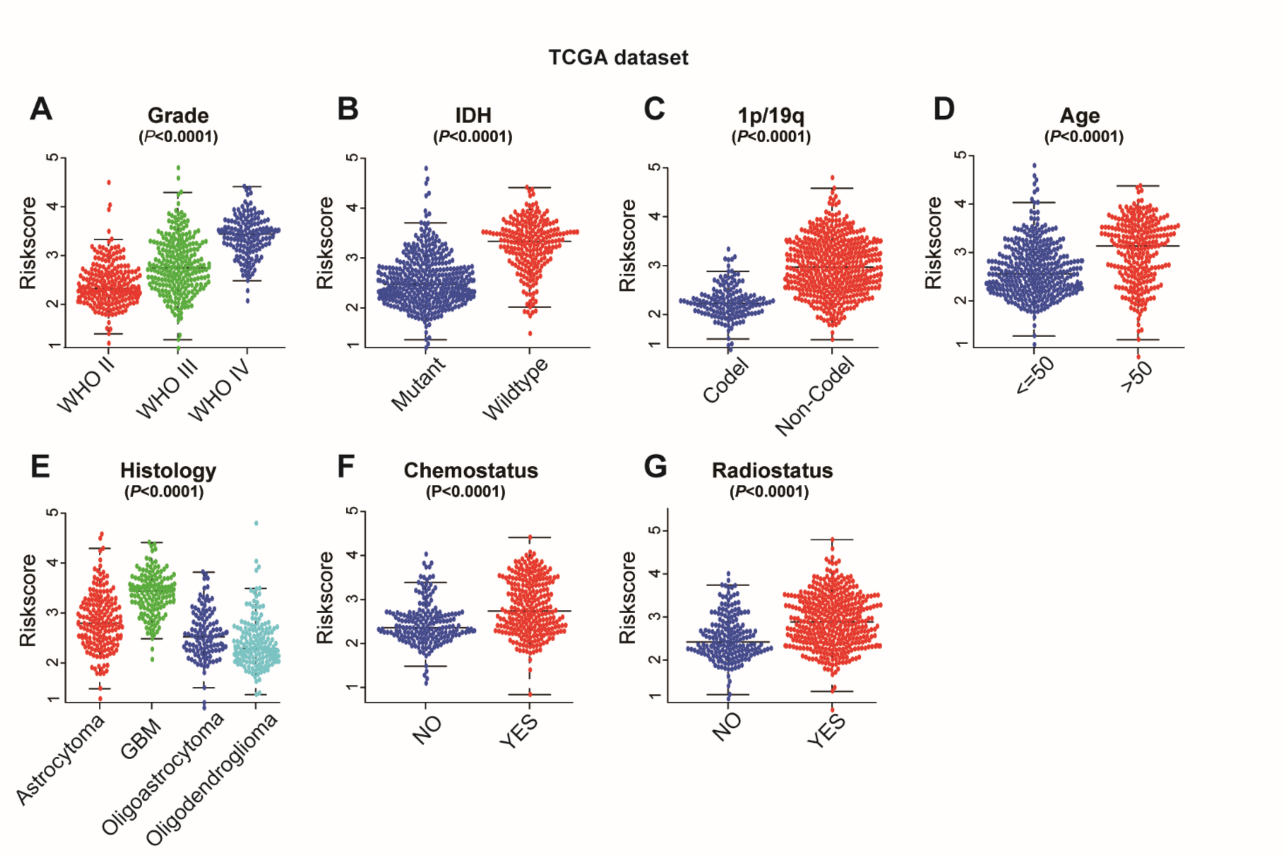

2.2 Supplementary Tables

Supplementary Table S1. The clinicopathological information of glioma patients in the CGGA and the TCGA datasets.

Characteristics		CGGA dataset	TCGA dataset
**Total patients**		306	616
**Gender**	Female	117	261
	Male	189	355
**Age**	>50	85	260
	<=50	221	356
**Grade**	II	97	221
	III	74	244
	IV	135	151
**Histology**	Oligodendroglioma	36	169
	Oligoastrocytoma	73	119
	Astrocytoma	62	177
	GBM	135	151
**IDH**	Mutant	167	387
	Wildtype	139	221
	NA	0	8
**1p/19q**	Codel	63	155
	Non-codel	239	457
	NA	4	4
**Chemostatus**	Yes	173	256
	No	118	185
	NA	15	175
**Radiostatus**	Yes	251	382
	No	44	184
	NA	11	50

Supplementary Table S2. The clinicopathological characteristics of glioma patients in three subtypes identified by selected RNA:m^{5}C methyltransferases with the CGGA dataset.

Characteristics		MC1	MC2	MC3	*P*-value
**Total patients**		104	120	82	
**Gender**	Female	41	38	38	**0.104**
	Male	63	82	44	
**Age**		42(17-70)	47(8-79)	38(19-61)	**<0.0001**
**Grade**	II	61	7	29	**<0.0001**
	III	25	26	23	
	IV	18	87	30	
**Histology**	Oligodendroglioma	26	1	9	**<0.0001**
	Oligoastrocytoma	33	18	22	
	Astrocytoma	27	14	21	
	GBM	18	87	30	
**IDH**	Mutant	70	20	77	**<0.0001**
	Wildtype	34	100	5	
**1p19q**	Codel	42	5	16	**<0.0001**
	Non-codel	60	115	64	
	NA	2	0	2	
**Chemostatus**	Yes	47	80	46	**0.006**
	No	51	35	32	
	NA	6	5		
**Radiostatus**	Yes	88	96	67	**0.602**
	No	12	19	13	
	NA	4	5	2	

Significance: $P^{*}<0.05$, $P^{**}<0.01$, $P^{***}<0.001$.

Supplementary Table S3. The clinicopathological characteristics of glioma patients with low-risk and high-risk groups in CGGA dataset

**Characteristics**		**CGGA dataset**			**TCGA dataset**		
		**low-risk**	**high-risk**	***P*-value**	**low-risk**	**high-risk**	***P*-value**
**Total patients**		153	153		308	308	
**Gender**	Female	62	55	0.41	136	125	**0.37**
	Male	91	98		172	183	
**Age**	>50	26	59	<0.0001	92	168	**<0.0001**
	<=50	127	94		216	140	
**Grade**	II	82	15	**<0.0001**	178	43	**<0.0001**
	III	38	36		119	125	
	IV	33	102		11	140	
**Histology**	Oligodendroglioma	2	34	**<0.0001**	137	32	**<0.0001**
	Oligoastrocytoma	20	53		81	38	
	Astrocytoma	29	33		79	140	
	GBM	102	33		11	98	
**IDH**	Mutant	112	55	**<0.0001**	268	119	**<0.0001**
	Wildtype	41	98		36	185	
	NA	0	0		4	4	
**1p/19q**	Codel	63	0	**<0.0001**	141	14	**<0.0001**
	Non-codel	88	151		166	291	
	NA	2	2		1	3	
**Chemostatus**	Yes	76	97	**0.01**	128	128	**<0.0001**
	No	70	48		151	34	
	NA	7	8		29	146	
**Radiostatus**	Yes	129	122	**0.3**	163	219	**<0.0001**
	No	19	25		130	54	
	NA	5	6		15	35	

Note: Significance: $P^{*}<0.05$, $P^{**}<0.01$, $P^{***}<0.001$.

Supplementary Table S4. The somatic mutation data of seven RNA:m^{5}C methyltransferases from the TCGA dataset.

Symbol	Gene Id	Chromo some	Variant Classification	Variant Type	Tumor Sample	Mutation Status
**NOP2**	4839	chr12	Missense_Mutation	SNP	TCGA-02-2485-01	Somatic
**NSUN2**	54888	chr5	Missense_Mutation	SNP	TCGA-76-4926-01	Somatic
**NSUN5**	55695	chr7	Missense_Mutation	SNP	TCGA-DU-A5TR-01	Somatic
**NSUN5**	55695	chr7	Translation_Start_Site	SNP	TCGA-76-4926-01	Somatic
**NSUN5**	55695	chr7	Missense_Mutation	SNP	TCGA-06-0744-01	Somatic
**NSUN7**	79730	chr4	Nonsense_Mutation	SNP	TCGA-02-2486-01	Somatic
